# The length of lateral radiographs significantly impacts the measurement of the femoral intramedullary axis in patients undergoing total knee arthroplasty

**DOI:** 10.1186/s42836-025-00350-x

**Published:** 2026-01-07

**Authors:** Moses K. D. El Kayali, Luis V. Bürck, Stephen Fahy, Lorenz Pichler

**Affiliations:** 1 Charité – Universitätsmedizin Berlin, Center for Musculoskeletal Surgery, 10117 Berlin, Germany; 2 Institute for Clinical and Experimental Surgery, Saarland University, 66421, Homburg/Saar, Germany; 3 Sporthopaedicum Berlin, 10627 Berlin, Germany; 4 Department of Orthopedics and Trauma-Surgery, Medical University of Vienna, 1090 Vienna, Austria

**Keywords:** Total knee arthroplasty, TKA, Sagittal alignment, Femoral component flexion, Short-segmented radiographs

## Abstract

**Background:**

Accurate femoral component alignment in the sagittal plane is crucial for total knee arthroplasty (TKA) success. In manual TKA, sagittal alignment is typically guided by the intramedullary axis (IMA) determined on lateral radiographs. However, due to femoral bowing, the IMA varies along the femoral shaft, raising the question of the optimal level for referencing this axis. As short-segmented knee radiographs (SSKR) are increasingly used in clinical practice, it is unclear whether they introduce systemic deviations in IMA determination. This study aimed to compare the IMA derived from SSKR and conventional lateral radiographs (CLR), and to assess whether axis deviation increases with femoral shaft length.

**Methods:**

This retrospective analysis included 153 patients undergoing primary TKA. The femoral IMA was determined using a two-circle method on both the full CLR and a 12.5 cm distal segment simulating SSKR. For the CLR axis, one circle was positioned at the most proximal point of the femoral shaft visible on the radiograph, and the second circle was placed 5 cm proximal to the distal femoral joint line. For the SSKR-based axis, the distal circle remained identical, while the proximal circle was repositioned 12.5 cm proximal to the joint line. Measurements were performed twice by two observers. The angular deviation between CLR- and SSKR-based axes was reported in degrees. A one-sample *t*-test was used to test for statistical significance. Clinically relevant deviation was defined as ≥ 2°, and the number and percentage of such outlier cases were reported. Correlation between femoral shaft length and angular deviation was analyzed using Pearson correlation. A multivariable regression tested whether femoral length independently predicted angular deviation after adjusting for age, sex, BMI, and side.

**Results:**

The IMA on SSKR was significantly more posterior than on CLR, with a mean angular deviation of 2.3° ± 1.1° (95% CI: 2.2–2.5; *P* < 0.001; Cohen’s *d* = 2.1). In 57 cases (38%), deviation exceeded the clinically relevant threshold of ≥ 2°. A significant positive correlation was found between the visible femoral shaft length and the angular deviation between CLR and SSKR axes (*r* = 0.504, *P* < 0.001). In multivariable regression, femoral length remained an independent predictor of angular deviation after adjustment for age, sex, BMI, and side (*P* < 0.001).

**Conclusion:**

Referencing the IMA on SSKR results in a significantly more posterior axis compared to CLR, which may lead to increased femoral component flexion in TKA. Given the high incidence of outlier cases and their association with femoral shaft length, surgeons should be cautious when relying on short radiographs for preoperative planning of sagittal femoral alignment.

Video Abstract

**Supplementary Information:**

The online version contains supplementary material available at 10.1186/s42836-025-00350-x.

## Introduction

Total knee arthroplasty (TKA) is a well-established and effective surgical treatment for end-stage knee osteoarthritis, offering substantial improvements in pain, function, and quality of life [1]. However, up to 20% of patients remain dissatisfied with their outcomes despite advances in implant design, surgical technique, and perioperative care [2–4]. While dissatisfaction is likely multifactorial, suboptimal implant positioning and lower limb malalignment are recognized contributors to poor clinical outcomes [5–9].

Although coronal alignment has been extensively studied, sagittal alignment has received comparatively less attention [10]. Yet, even small deviations in sagittal femoral component positioning can significantly affect knee kinematics, soft tissue balance, and implant longevity [11–13].

Optimal sagittal alignment requires avoiding both excessive flexion and extension of the femoral component. Increased flexion may lead to flexion–extension gap asymmetry, while excessive extension raises the risk of anterior cortical notching [14–17]. Both malalignments can result in clinical issues such as a limited range of motion, instability, pain, or premature polyethylene wear [18–20].

In manual TKA, sagittal femoral alignment is typically guided by referencing the intramedullary (IM) canal, which is preoperatively assessed on lateral knee radiographs [21]. Inaccurate determination of the intramedullary axis (IMA) may result in a suboptimal entry point, potentially compromising the trajectory of the IM guide and leading to unintended femoral component malalignment. However, due to natural femoral bowing, the orientation of the IMA changes along the shaft, raising the question of where along the femur this axis should be referenced [22, 23]. This issue becomes increasingly relevant as short-segmented knee radiographs (SSKR) are more commonly used in clinical settings to reduce imaging time and radiation exposure. To date, it remains unclear whether referencing the IMA on SSKRs introduces systematic deviations compared to conventional lateral radiographs (CLR).

Given the influence of femoral component flexion–extension alignment on TKA balance and kinematics, the purpose of this study was to assess differences in the femoral IMA between SSKRs and CLRs. We hypothesized that (1) SSKR-based measurements would result in a significantly different IMA compared to CLR, and (2) the magnitude of this deviation would increase with greater femoral length visible on CLR.

## Methods

### Patients

This study was approved by the institutional ethics committee (approval number Nr. EA2/016/21) and conducted in accordance with the Declaration of Helsinki.

In this retrospective study, 398 patients who underwent TKA at our high-volume academic orthopaedic center between March 2021 and March 2023 were screened for eligibility. Inclusion criteria were the availability of a preoperative lateral knee radiograph with a minimum of 15 cm of visible femoral shaft, written informed consent for research participation, and complete clinical records.

**Fig. 1:**
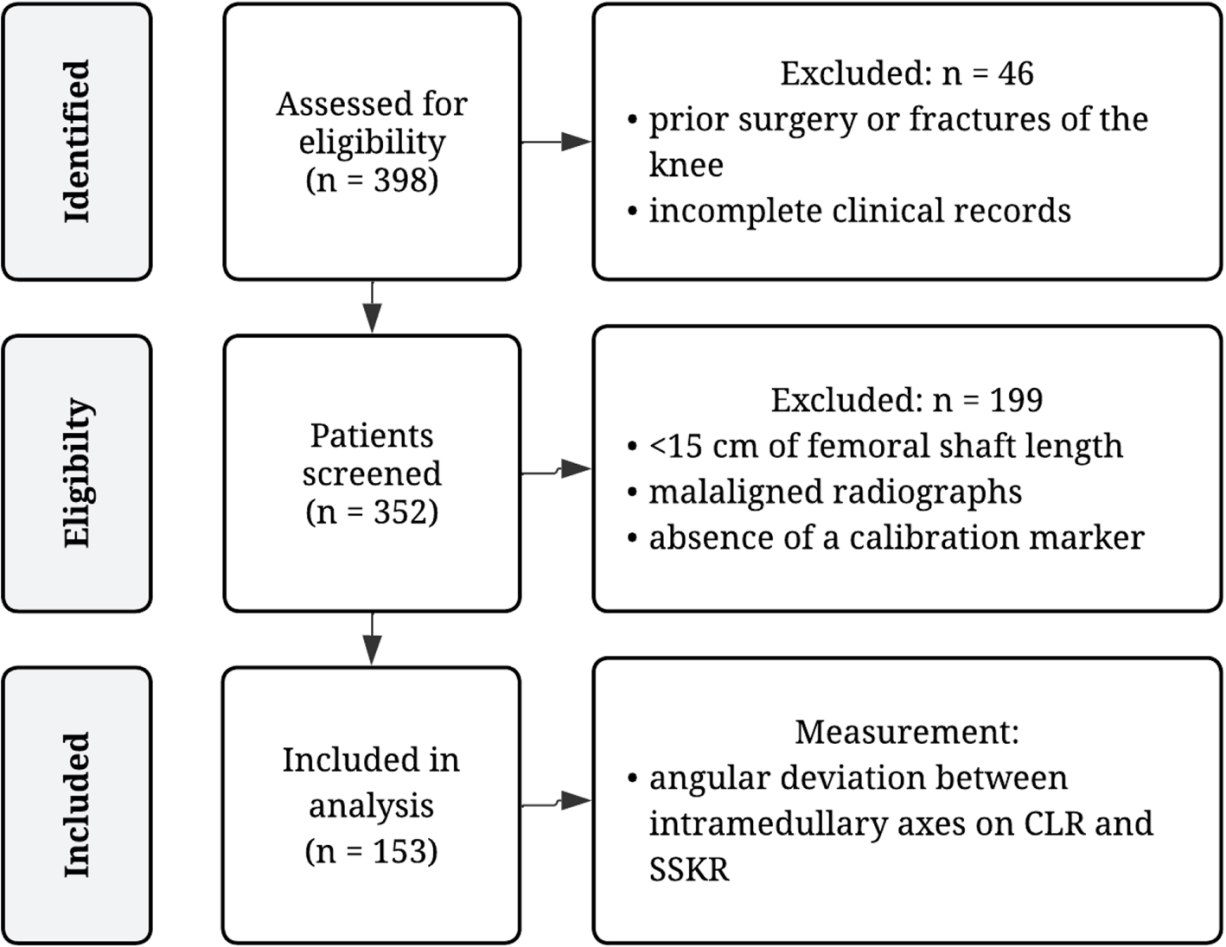
Flow chart of patient selection. *CLR* = *conventional lateral radiograph; SSKR* = *short-segmented knee radiograph*

Patients were excluded if lateral radiographs showed insufficient femoral shaft visualization (< 15 cm), malalignment in the form of rotation or abduction/adduction errors (defined by non-superimposed posterior and distal femoral condyles [24]), a history of prior surgery or fractures involving the affected knee, any bone-affecting metabolic disease or tumor, missing or incomplete patient records, absence of documented written informed consent, or lack of a calibration marker (reference ball) on the radiograph. The patient selection process is illustrated in Fig. 1.

Demographic data collected included age at the time of surgery, sex, body mass index (BMI), and side, which were obtained from the patients’ electronic medical records.

### Radiographs

Lateral, weight-bearing radiographs of the affected knee were obtained either during outpatient clinic visits or at the time of surgical planning for TKA. All radiographs were acquired using a standardized protocol: the knee was flexed to approximately 30°, the detector was aligned parallel to the sagittal plane, and the central X-ray beam was directed at the patellofemoral joint line.

Images were calibrated using a standard 25.4 mm (1-inch) radiographic reference marker. All radiographs were obtained using a digital radiography system (XGEO GC85A, Samsung, Seoul, South Korea).

### Measurement technique

**Fig. 2:**
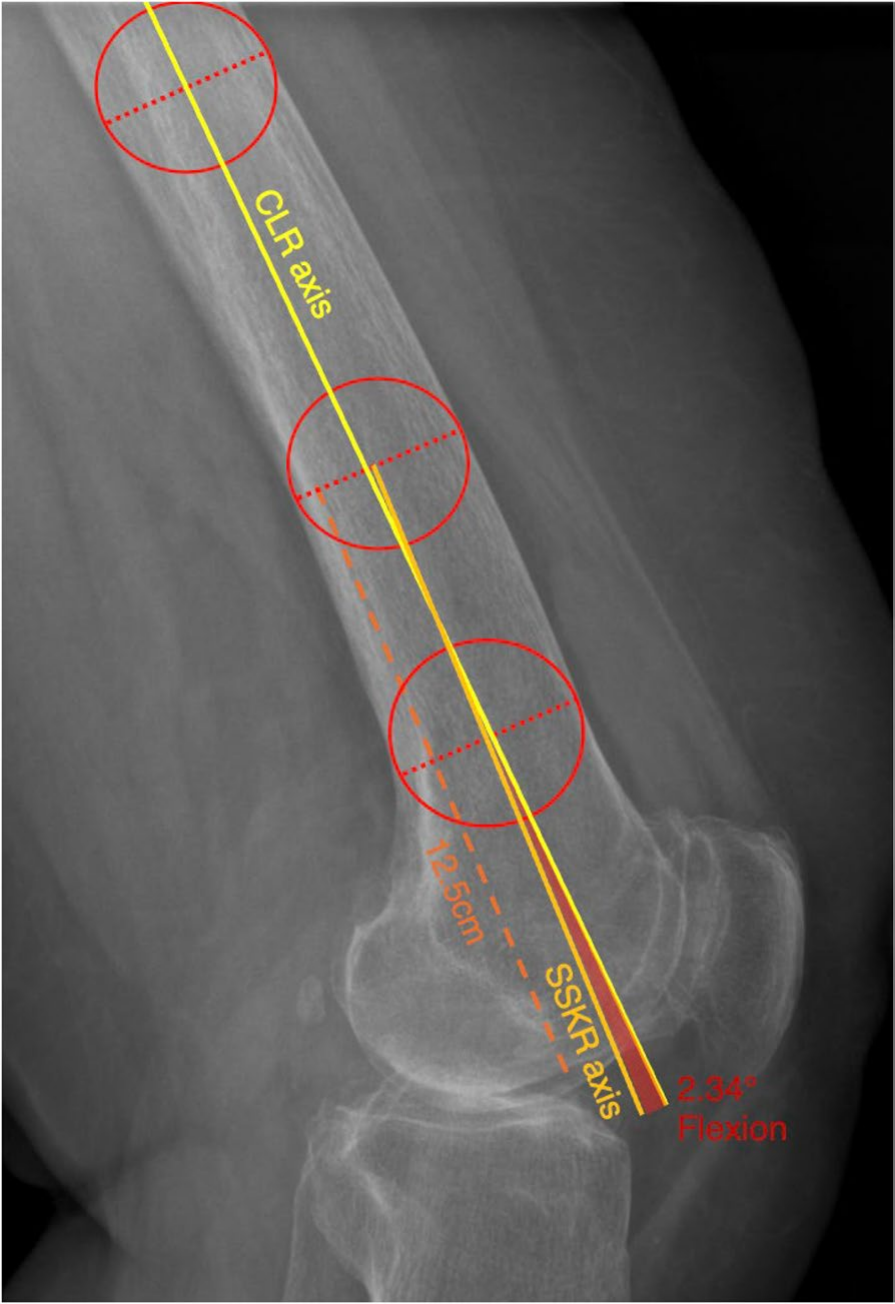
Determination of Intramedullary Axes. Lateral radiograph of a left knee demonstrating intramedullary axis determination on a CLR and on a 12.5 cm segment simulating an SSKR. The resulting angular difference (2.34°) is indicated to illustrate the measurement discrepancy between the two methods. *CLR* = *conventional lateral radiograph. SSKR* = *short-segmented knee radiograph*

In all CLRs, the IM femoral axis was determined twice: first using the full available femoral shaft length and second using a 12.5 cm distal segment measurement proximally from the level of the distal femoral condyles, simulating an SSKR. The 12.5 cm segment was chosen to approximate the typical 10–15 cm field of view of standard short lateral knee radiographs reported in previous comparative studies [25–30]. The IMA was defined as the line connecting the centers of two circles placed within the medullary canal, representing the midpoints of the femoral shaft at the respective proximal and distal locations [22]. Each circle was drawn using the PACS measurement tool and adjusted to best fit the outer cortical margins of the femoral shaft at the defined level, ensuring a symmetric fit around the bone contour. In cases with femoral curvature, circles were aligned perpendicular to the local shaft orientation at each respective level to follow the anatomical course of the femur. For the CLR axis, one circle was positioned at the most proximal point of the femoral shaft visible on the radiograph, and the second circle was placed 5 cm proximal to the distal femoral joint line. For the SSKR-based axis, the distal circle remained identical, while the proximal circle was repositioned 12.5 cm proximal to the joint line. The angular deviation between the SSKR- and CLR-based axes was measured in degrees, reported to two decimal places. The measurement technique is illustrated in Fig. 2.

All measurements were conducted using a PACS workstation (Centricity RIS-I 4.2 Plus, GE Healthcare, Chicago, IL, USA).

### Statistics

All extracted data were compiled and summarized using Microsoft Excel (version 16.78, Microsoft Corporation, Redmond, WA, USA). Statistical analysis was performed using IBM SPSS Statistics (Version 28.0, IBM Corp., Armonk, NY, USA).

Descriptive statistics were reported as means, standard deviations, and 95% CI. The Shapiro–Wilk test was used to assess the normality of continuous variables, and Levene’s test was applied to evaluate the homogeneity of variances. For normally distributed data, comparisons between groups were performed using an independent-sample *t*-test. In cases of non-normal distribution, the Mann–Whitney U test was used. Statistical significance was defined as a *p* *P *< 0.05 for all comparisons.

To test the primary hypothesis that the angular deviation between the SSKR and CLR IMA differed from zero, a two-tailed one-sample t-test was performed.

The angular deviation between the short-segment and conventional intramedullary axes was reported in degrees to two decimal places. A threshold of ≥ 2° angular deviation was defined as clinically relevant, based on previously reported accuracy limits for component alignment in TKA [31]. The number and percentage of cases exceeding this threshold were reported as outliers. To test the hypothesis that femoral shaft length influenced the angular deviation between short-segment and conventional radiographs, Pearson correlation analysis was performed for normally distributed data, and Spearman correlation was used otherwise. For interpretation of effect size, the Pearson correlation coefficient (*r*) was defined as „small “(≥ 0.1), „moderate “(≥ 0.3), and „high “(≥ 0.5) [32]. Correlation coefficients (*r*) and corresponding p-values were reported. To test whether the association between femoral shaft length and angular deviation was independent of patient characteristics, a multivariable linear regression analysis was performed. Angular deviation served as the dependent variable, and femoral shaft length, age, sex, BMI, and side (left/right) were entered as predictors. Regression coefficients (*β*), 95% confidence intervals (CI), and *P*-values were reported.

To determine the adequacy of the sample size, a post-hoc power analysis was conducted using G*Power [33] (Version 3.1.9.6, Heinrich Heine University, Düsseldorf, Germany). Based on the observed effect size (Cohen’s *d* = 2.1), a two-tailed one-sample t-test with an alpha level of 0.05 and a sample size of 153 achieved a statistical power of > 0.99.

To evaluate measurement reliability, two independent observers, both orthopaedic surgery residents (M.E. & L.P.) with extensive training in arthroplasty and expertise in preoperative planning, each performed all measurements at two separate time points, with a minimum interval of 14 days. Measurements were conducted under the supervision of a radiologist specialized in musculoskeletal imaging. Radiographs were analyzed in random order, and both observers were blinded to each other’s results and to their own previous measurements. Inter- and intra-rater reliability were assessed using the intraclass correlation coefficient (ICC), calculated based on a two-way random effects model with absolute agreement. ICC values were interpreted as follows: poor (< 0.5), moderate (0.5–0.75), good (0.75–0.9), and excellent (> 0.9) [34]. Inter-rater reliability for angular deviation measurements was excellent, with an ICC(2,1) of 0.92 (95% CI, 0.89–0.95). Intra-rater reliability was similarly high, with ICCs of 0.91 (95% CI, 0.87–0.94) for observer 1 (M.E.) and 0.92 (95% CI, 0.88–0.95) for observer 2 (L.P.).

## Results

A total of 153 lateral knee radiographs met the inclusion criteria, comprising 91 (59%) male and 62 (41%) female patients. Demographic and clinical characteristics are summarized in Table 1.

**Table 1:** Patient demographics

Parameter	Mean ± SD or no. (%)
Age, years	73.41 ± 9.8
BMI	29.81 ± 5.0
Sex	
Male	91 (59%)
Female	62 (41%)
Side	
Right	75 (50%)
Left	78(50%)

*SD* = *standard deviation; BMI* = *body mass index*

The mean femoral length measured on CLR was 18.1 cm ± 2.4 cm (95% CI: 17.7–18.4). The IMA determined on SSKR showed a significantly more posterior orientation (flexion position) compared to CLR, with a mean angular deviation of 2.3° ± 1.1° (95% CI: 2.2–2.5; one-sample *t*-test, *P* < 0.001; Cohen’s *d* = 2.1).

In 58 cases (38%), the angular deviation exceeded the clinically relevant cut-off of 2°.

A statistically significant high positive correlation was observed between femoral length and angular deviation (*r* = 0.503, *P* < 0.001). In the multivariable regression model, femoral length remained an independent predictor of angular deviation (*β* = 0.164° per cm; 95% CI, 0.086–0.242; *P* < 0.001), whereas age (*P* = 0.21), sex (*P* = 0.28), BMI (*P* = 0.68), and side (*P* = 0.51) were not significantly associated with angular deviation.

## Discussion

This study identified a consistent and statistically significant difference in the femoral IMA when measured on SSKR versus CLR. The axis measured on SSKR was located more posteriorly, resulting in a mean angular deviation of 2.3° ± 1.1° (*P* < 0.001; Cohen’s *d* = 2.1) and simulating increased femoral component flexion. These findings confirm our first hypothesis, demonstrating that SSKR radiographs introduce a measurable bias in IMA orientation. Furthermore, the magnitude of this posterior deviation correlated positively with femoral length (*r* = 0.504, *P* < 0.001), confirming our second hypothesis that a greater visible femoral length on CLR is associated with increased angular deviation when referencing a fixed 12.5 cm segment. Notably, 38% of cases exceeded the clinically relevant threshold of 2°.

**Fig. 3:**
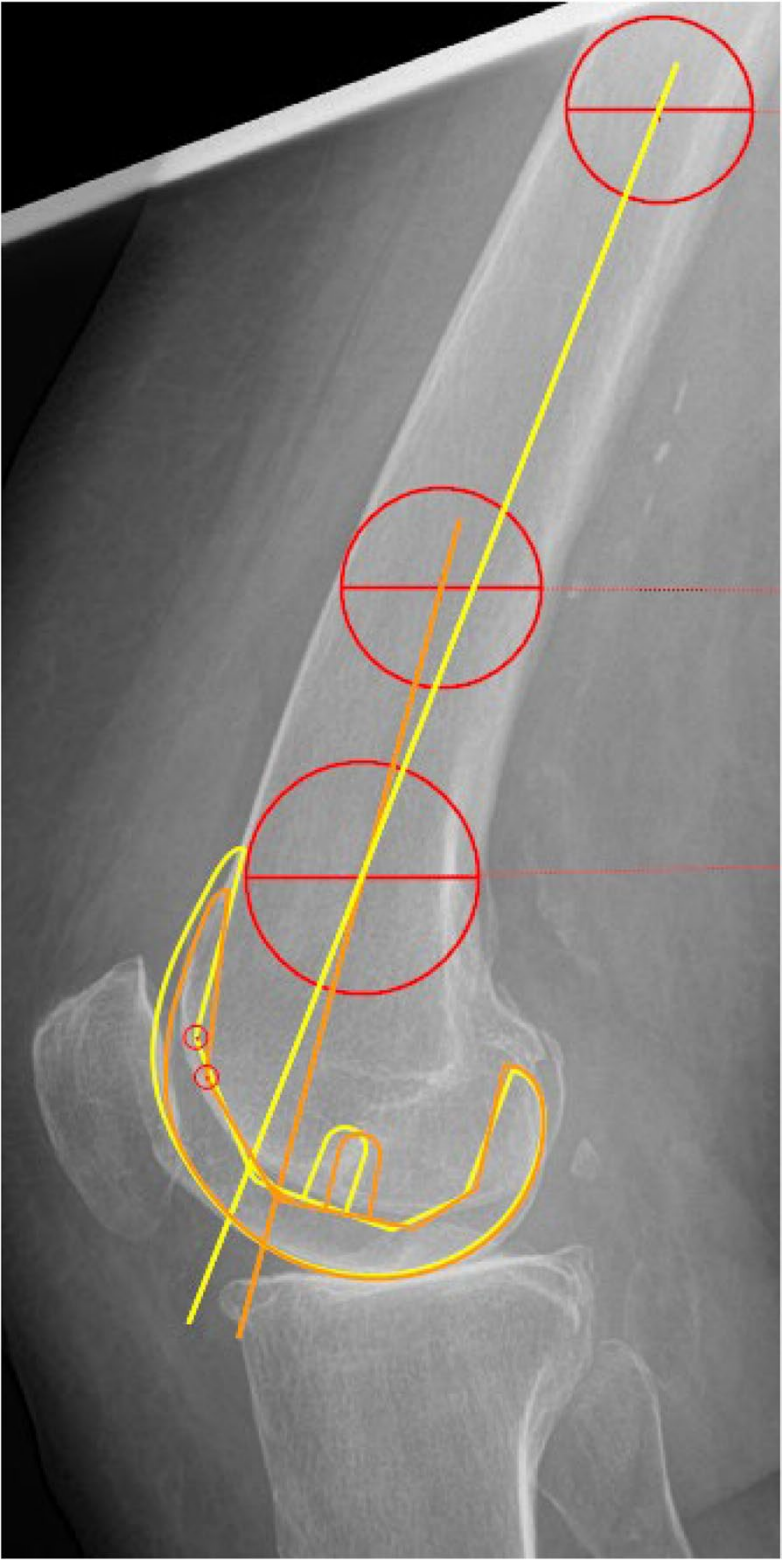
Femoral component positioning: CLR vs. SSKR-based IMA. Preoperative lateral radiograph depicting the IMA measured along the full femoral shaft on a CLR and on a 12.5 cm segment simulating an SSKR. Using two-dimensional digital templating with TraumaCad (Brainlab AG, Munich, Germany), femoral component positioning was simulated based on each axis, aiming for neutral sagittal alignment. The yellow outline illustrates the femoral component aligned to the CLR-based IMA, while the orange outline shows the SSKR-based positioning, demonstrating increased femoral component flexion resulting from the more posterior IMA on SSKR. *CLR* = *conventional lateral radiograph; SSKR* = *short-segmented knee radiograph; IMA* = *intramedullary axis*

This information is important for surgeons to consider when planning femoral component positioning based on SSKR, as these images may not accurately represent the sagittal IMA visible on CLR. In particular, the use of SSKR may lead to unintended increases in femoral component flexion. An example of the resulting differences in femoral component positioning when referencing the IMA from SSKR versus CLR is illustrated in Fig. 3.

The observed mean deviation of 2.3° toward increased femoral flexion when referencing the IMA on SSKR is not only statistically significant but may also have important clinical implications. Even small changes in sagittal femoral alignment can alter knee kinematics, soft tissue balance, and implant longevity [12, 13, 17]. Tsukeoka and Lee demonstrated that each additional 2° of femoral flexion reduces posterior medial condylar resection by approximately 1 mm [35]. When considering flexion and extension gaps, alterations in resected bone, as well as increased femoral flexion, can result in gap asymmetry [35–37]. Similarly, Matziolis et al. reported a 1-mm tightening of the flexion space with 1.6°–2.2° of femoral component flexion [19]. Such changes may manifest clinically as a limited range of motion, instability, pain, effusion, or abnormal polyethylene wear [18–20]. Accordingly, these findings highlight the importance of using adequately long radiographs or advanced imaging modalities to ensure accurate sagittal referencing.

Prior studies have demonstrated that IM referencing offers superior accuracy compared to extramedullary guiding systems in sagittal plane alignment [38, 39]. As a result, most surgeons continue to rely on IM alignment rods inserted through a distal femoral entry point. To aid in locating this entry point intraoperatively, visual cues such as Whiteside’s line [40] are often combined with preoperative lateral radiographs. Many factors can affect IM alignment accuracy, including the location of the rod entry point in the coronal plane, femoral canal diameter, and structural features of the rod [41–44]. As even minor deviations in the insertion point can result in malalignment by several degrees, determination of the ideal femoral entry point and orientation along the IMA is crucial [45, 46]. However, if the IMA is derived from an SSKR that does not reflect the full femoral shaft morphology, this may compromise the accuracy of the guide’s trajectory, with direct implications for component flexion/extension positioning. Our results show that the IMA measured on SSKR not only differed significantly from that measured on CLR and was consistently biased toward increased flexion, but in 38% of cases, the deviation would result in a clinically relevant flexion of the femoral component by 2°.

Despite the well-documented importance of coronal alignment in TKA, sagittal alignment remains comparatively underexplored [10]. For example, while current alignment strategies, including mechanical, kinematic, and functional alignment, each set specific targets for component position in the coronal plane, few of these strategies specify optimal sagittal alignment of the femoral component [47–49]. One of the key unresolved issues in this domain is the determination of the most appropriate reference axes for assessing sagittal femoral alignment, especially when conventional instrumentation such as IM guides is used. In contrast to the mechanical axis used in coronal planning, there is no universally accepted sagittal axis for guiding femoral component positioning or selecting the femoral entry point. As our findings suggest, even subtle variations in how and where the IMA is measured, particularly on SSKR, can lead to clinically meaningful changes in component alignment.

A recent cadaveric study comparing the IM and navigated femoral axes found that computer-navigated or robotic references resulted in an average of 1.4° less flexion when referencing the center of the femoral head rather than in the IM canal [50]. The authors also found that increased femoral bowing was significantly associated with larger discrepancies between the two axes (*r*^2^ = 0.7, *P* < 0.001). This is consistent with prior studies indicating that femoral bowing can significantly influence lower limb alignment when using IM guides, as the optimal entry point and trajectory of the IM rod becomes increasingly difficult to control in the presence of pronounced femoral bowing and less femoral neck anteversion [51, 52]. Although femoral bowing was not directly assessed in our study, the confirmation of our second hypothesis, that angular deviation increased with greater visible femoral shaft length on CLR, suggests that bowing likely contributes to this discrepancy, as it is underrepresented on SSKR.

Taken together, these results highlight that sagittal alignment is highly dependent on the chosen reference axis and individual anatomical characteristics. In particular, IM-based referencing from SSKR may introduce a consistent flexion bias. Given the current lack of a gold standard for assessing sagittal femoral alignment, these gaps in standardization underscore the need to re-evaluate how sagittal alignment is defined and incorporated into preoperative planning for TKA. In practical terms, imaging protocols for preoperative planning should ensure visualization of a sufficiently long femoral segment to allow accurate determination of the IMA, particularly in patients with pronounced femoral bowing. In such cases, advanced imaging or intraoperative navigation and robotic-assisted systems may provide more reliable sagittal alignment by compensating for anatomical curvature not captured on SSKR. If only SSKR is available, surgeons should anticipate an average flexion bias of approximately 2°, which tends to increase with shorter visible femoral shaft length.

## Limitations

This study has several limitations in addition to those inherent to its retrospective design. First, SSKR was simulated by digitally cropping CLR. This approach does not account for variations in beam centering, magnification, or patient positioning that occur during actual short-segment image acquisition. Therefore, while this method isolates the geometric effect of reduced femoral length, it may not fully replicate real clinical imaging conditions. Second, we did not assess anatomical characteristics such as femoral bowing, which may partly explain the angular deviations observed between IMA. Third, our study population was relatively homogeneous, consisting predominantly of Caucasian patients. Given that femoral bowing is more prevalent in Asian populations [53–55], the generalizability of our findings may be limited. Fourth, our analysis was based solely on two-dimensional radiographs, which, although commonly used in clinical practice, may not fully capture the complex three-dimensional (3D) anatomy of the femoral canal. Future studies using computed tomography or 3D reconstruction may provide a more accurate assessment of axis deviation relative to anatomical landmarks. Fifth, we were unable to correlate differences in sagittal alignment with clinical outcomes such as range of motion, pain, or patient satisfaction. While this was beyond the scope of the present study, future prospective research should investigate the clinical relevance of these radiographic deviations.

## Conclusion

In conclusion, referencing the IMA on SSKR results in a significantly more posterior axis compared to CLR. When the SSKR-derived IMA is used for preoperative planning, it may lead to a suboptimal entry point, altering the trajectory of the IM guide and potentially resulting in unintended femoral component flexion. Surgeons should be aware of this difference during both preoperative planning, especially when using conventional IM instrumentation, to ensure accurate femoral component positioning.

## Data Availability

The datasets generated and analyzed during the current study are available from the corresponding author upon reasonable request.

## Abbreviations

BMI: Body mass index
CLR: Conventional lateral radiographs
EM: Extramedullary
ICC: Intra-class correlation coefficient
IM: Intramedullary
IMA: Intramedullary axis
SSKR: Short-segmented knee radiographs
TKA: Total knee arthroplasty
